# Meta-analysis of data from spaceflight transcriptome experiments does not support the idea of a common bacterial “spaceflight response”

**DOI:** 10.1038/s41598-018-32818-z

**Published:** 2018-09-26

**Authors:** Michael D. Morrison, Wayne L. Nicholson

**Affiliations:** Department of Microbiology and Cell Science, University of Florida, Merritt Island, FL USA

## Abstract

Several studies have been undertaken with the goal of understanding how bacterial transcriptomes respond to the human spaceflight environment. However, these experiments have been conducted using a variety of organisms, media, culture conditions, and spaceflight hardware, and to date no cross-experiment analyses have been performed to uncover possible commonalities in their responses. In this study, eight bacterial transcriptome datasets deposited in NASA’s GeneLab Data System were standardized through a common bioinformatics pipeline then subjected to meta-analysis to identify among the datasets (i) individual genes which might be significantly differentially expressed, or (ii) gene sets which might be significantly enriched. Neither analysis resulted in identification of responses shared among all datasets. Principal Component Analysis of the data revealed that most of the variation in the datasets derived from differences in the experiments themselves.

## Introduction

Space travel inside human-rated spacecraft exposes living organisms to a number of stressors including microgravity, ionizing radiation, vibration, and altered atmospheric composition^1^. During the history of human spaceflight, many studies have been performed in low-Earth orbit within the protection of Earth’s magnetic field, to understand how spaceflight factors, particularly microgravity, affect macroscopic organisms. As a result we have a fairly detailed mechanistic understanding of how humans^2^, animals^3^, and plants^4^ respond when exposed to microgravity. In contrast, we have a relatively poor understanding of how single-celled microorganisms respond to the microgravity environment.

In order to understand how microbes sense and respond to stresses encountered during human spaceflight, a number of experiments have been conducted inside various human space habitats (e.g. Shuttle, Mir, ISS) using model microbial systems. In early studies, various phenotypic outputs from microbes grown in space were measured, such as: growth rate and yield; virulence; biofilm formation and architecture; and resistance to antibiotics or abiotic stresses, to name but a few [reviewed in^5–7^]. In more recent years, with the advent of the genomics and post-genomics revolutions in biology, there have been efforts to understand more fundamental molecular aspects of microbial spaceflight responses, by performing global-scale “-omics” analyses of the transcriptome, proteome, or metabolome of microbes cultivated in spaceflight. Such studies have yielded insights into the molecular responses of certain individual microbes to the spaceflight environment^8–11^, but a common “spaceflight response” has not emerged. Collectively the data rather seem to indicate that each individual organism mounts its own unique response to the microgravity environment; to date, however, this notion has not been subjected to rigorous testing.

Datasets from spaceflight biology experiments have been deposited into a common repository, NASA’s GeneLab Data System (GLDS) (genelab.nasa.gov). The GLDS contains a collection of -omics data obtained from NASA-funded spaceflight and ground-based spaceflight simulation experiments. The GLDS was designed to consolidate and archive such datasets in a publicly accessible manner to facilitate the testing of novel research questions and new hypotheses, beyond those for which the spaceflight experiments were originally conducted. Often, exposure of a microorganism to a particular environmental stress (heat, cold, high salt, etc.) will provoke a stereotypic and reproducible response to that particular exposure (i.e., heat-shock, cold-shock, osmotic-shock, etc. response) which can be visualized by alterations in the organism’s global pattern of transcription, i.e., its transcriptome. We therefore sought to investigate spaceflight-induced changes in bacterial transcriptomes with an eye toward identifying commonalities in their response to spaceflight exposure. Transcriptome datasets were collected from the GLDS and analyzed using a standardized bioinformatics pipeline to detect and quantify differential gene expression. The results from each dataset were then compared to identify possible effects from spaceflight as well as spaceflight-independent effects such as those arising from different hardware configurations or growth conditions. We report here that our meta-analysis of the current transcriptome datasets deposited in the GLDS failed to find any significant commonalities in the response of diverse bacteria to the spaceflight environment.

## Results

Examination of the data summarized in Table 1 revealed that the transcriptome datasets deposited in the GLDS were derived from spaceflight experiments utilizing different hardware, media, incubation times, and transcript measurement technologies. In all cases, flight (FL) cultures were compared with a set of ground control (GC) cultures incubated under matched conditions of hardware, growth medium, and temperature. Five different bacterial species were examined, 3 Gram-negative species (*P. aeruginosa, S. enterica*, and *R. rubrum*) and 2 Gram-positive species (*B. subtilis* and *S. aureus*). Two species (*P. aeruginosa* and *S. aureus*) have been flown only once, whereas 3 species (*B. subtilis, R. rubrum*, and *S. enterica*) have each been flown in space on two separate occasions (Table 1). The raw data from each experiment were extracted from the GLDS, converted into a common format, and analyzed using the bioinformatics pipeline as described in Methods.

**Table 1:** Bacterial transcriptome datasets used in this study.

GLDS	Mission	Organism	Medium	Hardware	Temp (°C)	Time	Platform	Reference
31	MESSAGE 2	*R. rubrum*	SPYA	Sealed Petri Dish	21	10 d	Microarray (GenePix)	^11^
39	BASE A	*R. rubrum*	SSA	6 well culture plate	21	12 d	Microarray (GenePix)	^11^
15	STS-115	*P. aeruginosa*	LB	FPA	23	25 h	Microarray (Affymetrix)	^8^
11	STS-115	*S. enterica*	LB	FPA	23	25 h	Microarray (QuantArray)	^9^
11	STS-123	*S. enterica*	M9	FPA	23	25 h	Microarray (QuantArray)	^10^
185	BRIC-21	*B. subtilis*	TSYG	BRIC-PDFU	23	25 h	RNA-seq (Illumina)	Morrison *et al*. 2018
138	BRIC-23	*B. subtilis*	TSYG	BRIC-PDFU	22	36 h	RNA-seq (Illumina)	^15^
145	BRIC-23	*S. aureus*	TSYG	BRIC-PDFU	22	48 h	RNA-seq (Illumina)	^15^

Abbreviations: BRIC-PDFU, Biological Research in Canisters-Petri Dish Fixation Unit; FPA, Fluid Processing Apparatus; LB, Lennox broth; M9, M9 minimal broth; SPYA, Sistrom peptone-yeast agar; SSA, Sistrom succinate agar; TSYG, trypticase soy yeast extract broth with 10% glycerol.

### Principal Component Analysis

**Figure 1:**
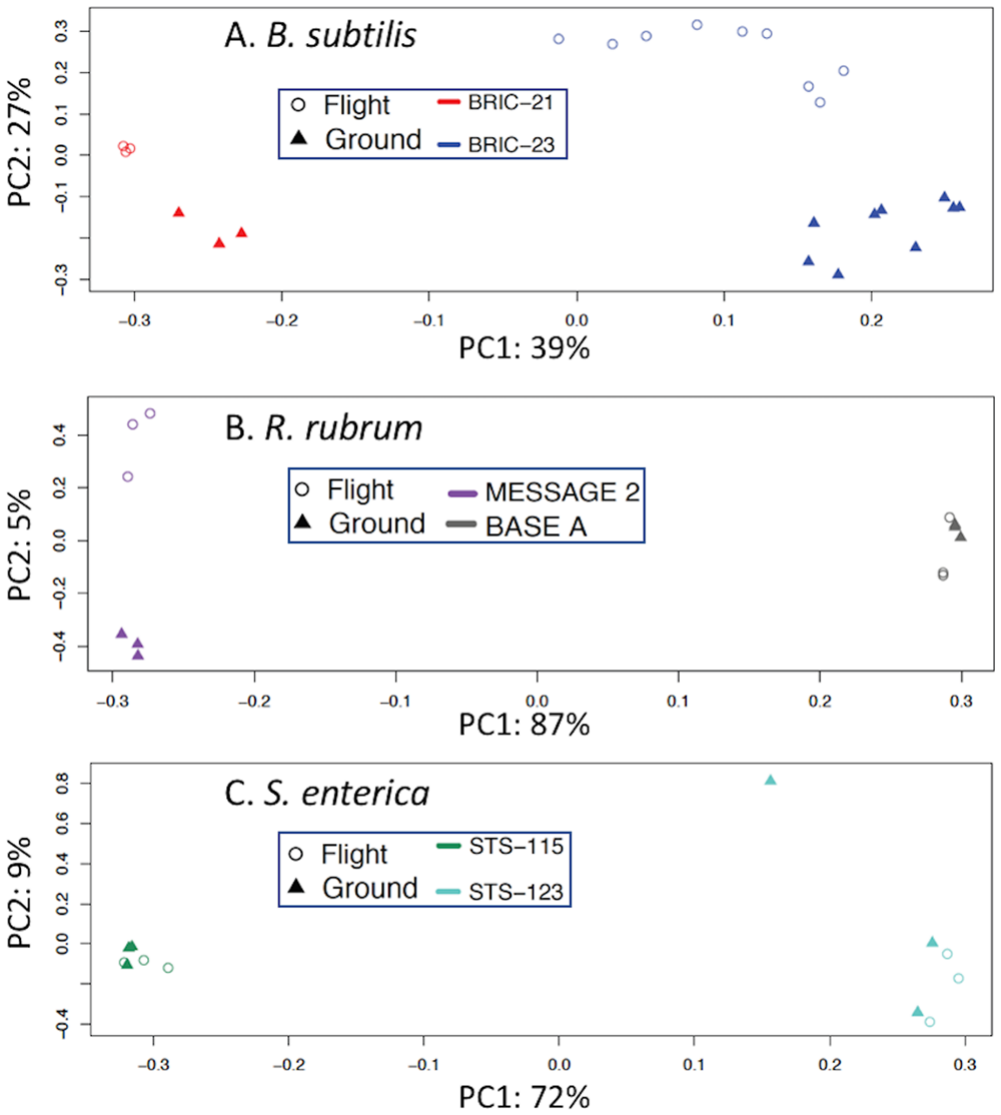
PCA plots of datasets for which 2 independent spaceflight experiments have been conducted. Datasets included are: (**A**) BRIC-21 and BRIC-23 (*B. subtilis*), (**B**) MESSAGE 2 and BASE A (*R. rubrum*) and (**C**). STS-115 and STS-123 (*S. enterica*).

Datasets for the 3 organisms flown on 2 separate occasions presented an opportunity to better understand the possible sources of variation among the datasets. We therefore performed Principal Component Analysis (PCA) and plotted the results (Fig. 1). Examination of the plots revealed some interesting features. First, the tightness of clustering of replicates within each experiment is an indication of reproducibility among the replicates. For example, in the *B. subtilis* data, BRIC-21 FL and GC samples clustered relatively tightly, whereas BRIC-23 FL and GC samples showed greater dispersion (Fig. 1A). The *R. rubrum* datasets showed reasonably tight clustering of replicates (Fig. 1B), as did the *S. enterica* datasets, with the exception of an outlier in the STS-123 GC replicates (Fig. 1C). Second, examination of Principal Component 1 (PC1) revealed that for all three organisms the greatest source of variation in the datasets was derived from differences in the two experimental trials (Fig. 1). Third, examination of Principal Component 2 (PC2) revealed variation between FL and GC samples within the same experiment. Examination of PC2 showed that in the BRIC-21, BRIC-23 (*B. subtilis*), and MESSAGE 2 (*R. rubrum*) datasets the FL and GC samples formed well-separated groups, indicating differences in gene expression patterns (Fig. 1A and B). In contrast, FL vs. GC samples in the BASE A (*R. rubrum*), STS-115 and STS-123 (*S. enterica*) experiments clustered closely together, indicating very little difference in gene expression patterns between FL and GC samples from these missions (Fig. 1B and C).

### Differential Expression Analysis: Gram-negative species

The Gram-negative species examined included the α-proteobacterium *R. rubrum*, and two γ-proteobacteria, *P. aeruginosa* and *S. enterica*; the raw data for these organisms were originally derived from fluorescence microarray technology using three different platforms (GenePix, Affymetrix, and QuantArray) (Table 1). In order to make meaningful comparisons among the datasets, they were imported into a common bioinformatics pipeline as described in Methods and analyzed to discover significantly differentially expressed transcripts.

*P. aeruginosa*. Crabbé *et al*.^8^ cultivated *P. aeruginosa* strain PAO1 in Lennox broth in Fluid Processing Apparatus (FPA) hardware on the STS-115 mission. They reported the differential expression of 167 genes between FL and GC samples (52 up-regulated and 115 down-regulated in FL samples)^8^. After the raw datasets from that experiment were run through our bioinformatics pipeline and the resulting *P* values adjusted for multiple testing bias^12^, we found no significantly differentially expressed transcripts between FL vs. GC samples from that experiment (Table 2).

**Table 2:** Number of differentially expressed genes identified in each GLDS dataset.

Dataset	Genes up-regulated in FL	Genes down-regulated in FL
MESSAGE 2 (*R. rubrum*)	191	29
BASE A (*R. rubrum*)	41	9
STS-115 (*P. aeruginosa*)	0	0
STS-115 (*S. enterica*)	6	98
STS-123 (*S. enterica*)	1	0
BRIC-21 (*B. subtilis*)	198	311
BRIC-23 (*B. subtilis*)	181	137
BRIC-23 (*S. aureus*)	161	50

*S. enterica*. Wilson *et al*.^9,10^ reported on the transcriptomic response to spaceflight of *S. enterica* serovar Typhimurium strain χ3339 on two separate missions, STS-115^9^ and STS-123^10^. In the STS-115 experiment, cells were cultivated in liquid Lennox broth in FPA hardware, and 167 transcripts were reported to be significantly differentially expressed (69 up-regulated and 98 down-regulated in FL samples)^9^. Processing of the raw data from this experiment through our bioinformatics pipeline resulted in identification of fewer (104 total) differentially expressed transcripts, and most of the discrepancy resided in genes up-regulated in FL samples (6 up- and 98-down regulated in FL samples) (Table 2). In the STS-123 experiment^10^, cells were cultivated in liquid M9 medium in FPA hardware, and 38 total transcripts were reported to be significantly differentially expressed (14 up-regulated and 24 down-regulated in FL samples)^10^. However, our processing and analysis of the raw data from this experiment resulted in identification only 1 significantly up-regulated transcript in FL samples and 0 significantly down-regulated transcripts (Table 2).

*R. rubrum*. Mastroleo *et al*.^11^ reported on the transcriptomic response to spaceflight of *R. rubrum* strain S1H on two separate experiments called MESSAGE 2 and BASE A. In the MESSAGE 2 experiment, *R. rubrum* was cultivated on Sistrom-peptone-yeast agar in Petri plates for 8 days, and 218 total transcripts were reported to be significantly differentially expressed (191 up-regulated and 27 down-regulated in FL samples)^11^. Our own processing of the raw data from the MESSAGE 2 experiment resulted in close agreement; we found 220 total transcripts differentially expressed (191 up- and 29 down-regulated in FL samples) (Table 2). In the BASE A experiment, *R. rubrum* was cultivated on Sistrom-succinate agar in Petri plates for 12 days, and 64 total transcripts were reported to be significantly differentially expressed (53 up-regulated and 11 down-regulated in FL samples)^11^. Our own processing of the raw data from the BASE A experiment resulted in reasonable agreement; we identified 50 differentially expressed genes (41 up-regulated and 9 down regulated in FL samples) (Table 2).

**Figure 2:**
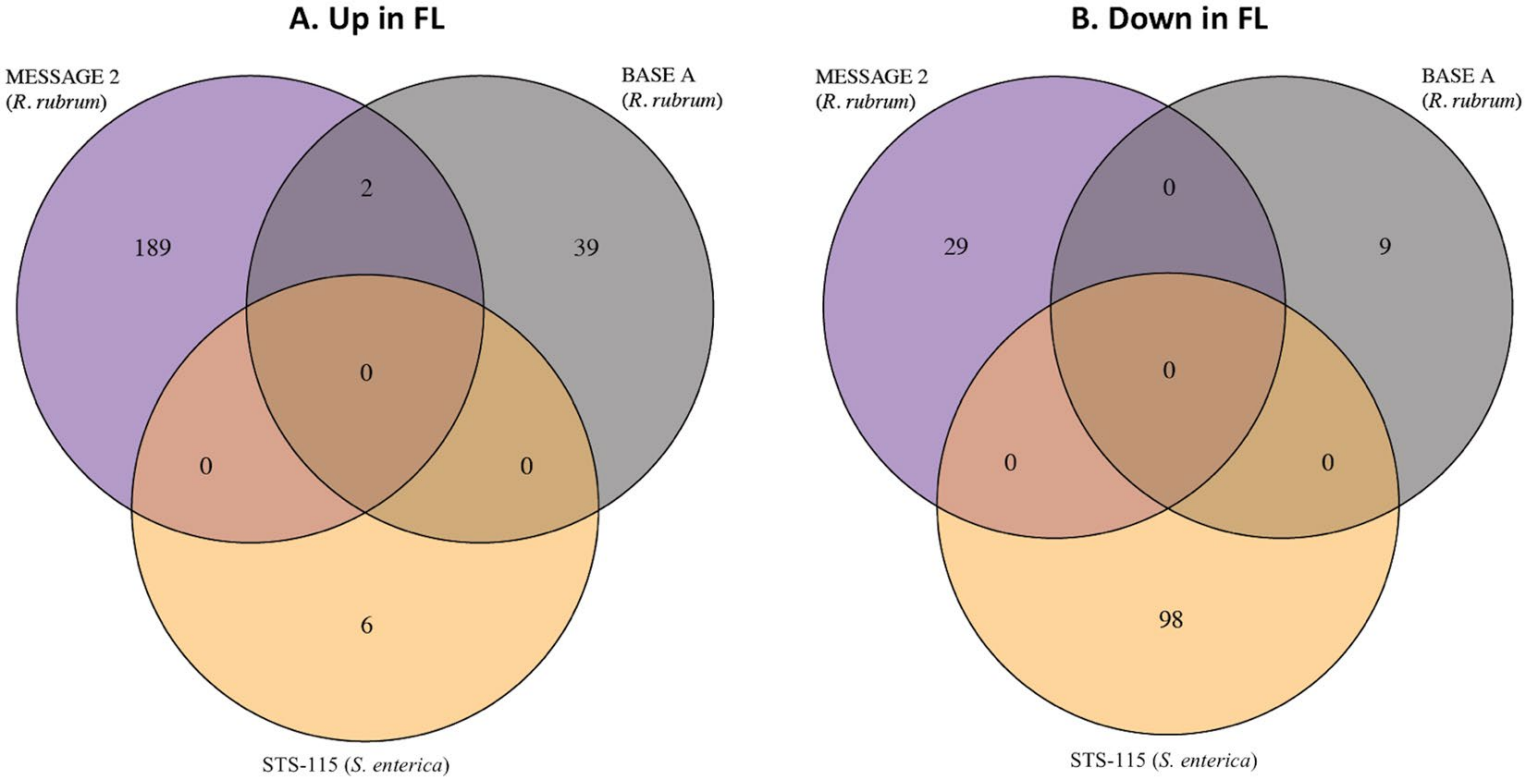
Venn diagrams depicting genes found to be up-regulated (**A**) or down-regulated (**B**) in common among the MESSAGE 2, BASE A, (both *R. rubrum*) and STS-115 (*S. enterica*) spaceflight datasets.

**Figure 3:**
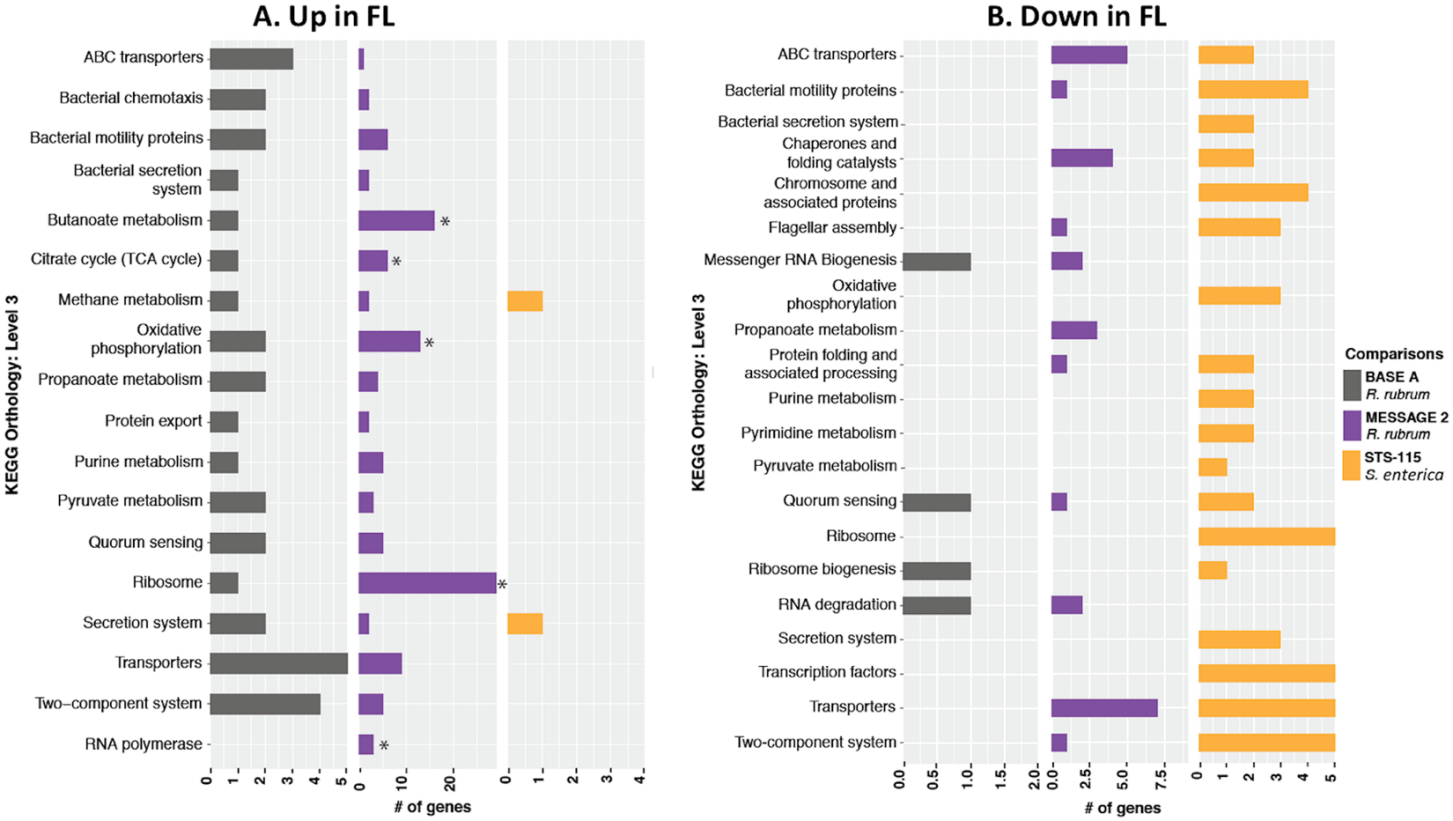
Functional characterization of the differentially expressed genes identified in the datasets for Gram-negative organisms. Depicted are numbers of genes belonging to the indicated biological pathways (KEGG Orthologies) found to up-regulated (**A**) or down-regulated (**B**) in FL samples from the *R. rubrum* BASE A and MESSAGE 2 experiments (gray and purple bars, respectively), and the *S. enterica* STS-115 experiment (yellow bars) experiment. Asterisks denote pathways deemed to be significantly enriched (adjusted *P* < 0.05, Fisher’s exact test).

We next compared the *S. enterica* STS-115 and the *R. rubrum* MESSAGE 2 and BASE A datasets using KEGG Orthology (KO) terms to identify differentially expressed genes in common. As depicted in the resulting Venn diagrams (Fig. 2), we failed to find any genes in common among the up-regulated (Fig. 2A) or down-regulated (Fig. 2B) transcripts from all three experiments, and in pairwise comparisons only 2 genes were found to be up-regulated in common, both from the *R. rubrum* MESSAGE 2 and BASE A experiments (Fig. 2A). The first of these genes belonged to KEGG Orthology K00341 and was annotated as *nuoL*, encoding NADH-quinone oxidoreductase subunit L, which shuttles electrons from NADH to quinones in the respiratory electron transport chain^13^. The second gene belonged to KEGG Orthology K02897 and was annotated as *rplY* encoding large subunit ribosomal protein L25^14^ (Table 3). In all other cases, genes were either significantly expressed but in the opposite direction or were not significantly differentially expressed (Table 3). Functional enrichment analysis of the differentially expressed genes found statistically significant enrichment of 4 up-regulated pathways from only one dataset (*R. rubrum* MESSAGE 2)—including butanoate metabolism, TCA cycle, oxidative phosphorylation, and ribosomes (denoted by asterisks in Fig. 3A). Among down-regulated pathways, no significantly enriched pathways were found (Fig. 3B).

**Table 3:** KEGG Orthology (KO) numbers identified as differentially expressed in two or more datasets in Gram-negative organisms*.

KO	*R. rubrum*				*S. enterica*				Description
	MESSAGE 2		BASE A		STS-115		STS-123		
	L2FC	*P* value	L2FC	*P* value	L2FC	*P* value	L2FC	*P* value	
N/A	**2.11**	**4.25E-07**	**−1.17**	**2.45E-02**	—	—	—	—	UPF0391 membrane protein
K00341	**1.13**	**8.03E-07**	**1.61**	**7.98E-04**	0.41	5.39E-01	−0.52	5.63E-02	NADH-quinone oxidoreductase subunit L
N/A	**2.25**	**5.56E-10**	**−1.23**	**1.24E-02**	—	—	—	—	Uncharacterized protein
K02897	**1.49**	**1.63E-07**	**1.39**	**1.97E-03**	−0.65	4.94E-01	−0.24	9.62E-01	large subunit ribosomal protein L25
K02078	**1.49**	**4.56E-07**	−0.41	1.24E-03	**−2.76**	**4.56E-02**	−0.24	9.13E-01	acyl carrier protein
K00242	**1.33**	**3.98E-07**	−0.01	9.64E-01	**−1.62**	**4.81E-02**	0.25	9.53E-01	succinate dehydrogenase/ fumarate reductase, membrane anchor subunit
K04047	**1.20**	**5.74E-06**	−0.19	4.15E-01	**−1.60**	**2.71E-02**	−0.11	9.79E-01	starvation-inducible DNA-binding protein
K02911	**1.30**	**6.80E-08**	0.34	9.46E-03	**−2.87**	**4.49E-02**	0.20	9.38E-01	large subunit ribosomal protein L32
K02892	**1.52**	**7.44E-09**	0.52	3.89E-03	**−2.99**	**3.48E-02**	−0.04	9.86E-01	large subunit ribosomal protein L23
K02863	**1.03**	**1.03E-06**	0.09	4.57E-01	**−2.62**	**2.71E-02**	0.60	7.79E-01	large subunit ribosomal protein L1

*Significantly differentially expressed transcripts are denoted in boldface type. N/A, not applicable.

### Differential Expression Analysis: Gram-positive species

**Figure 4:**
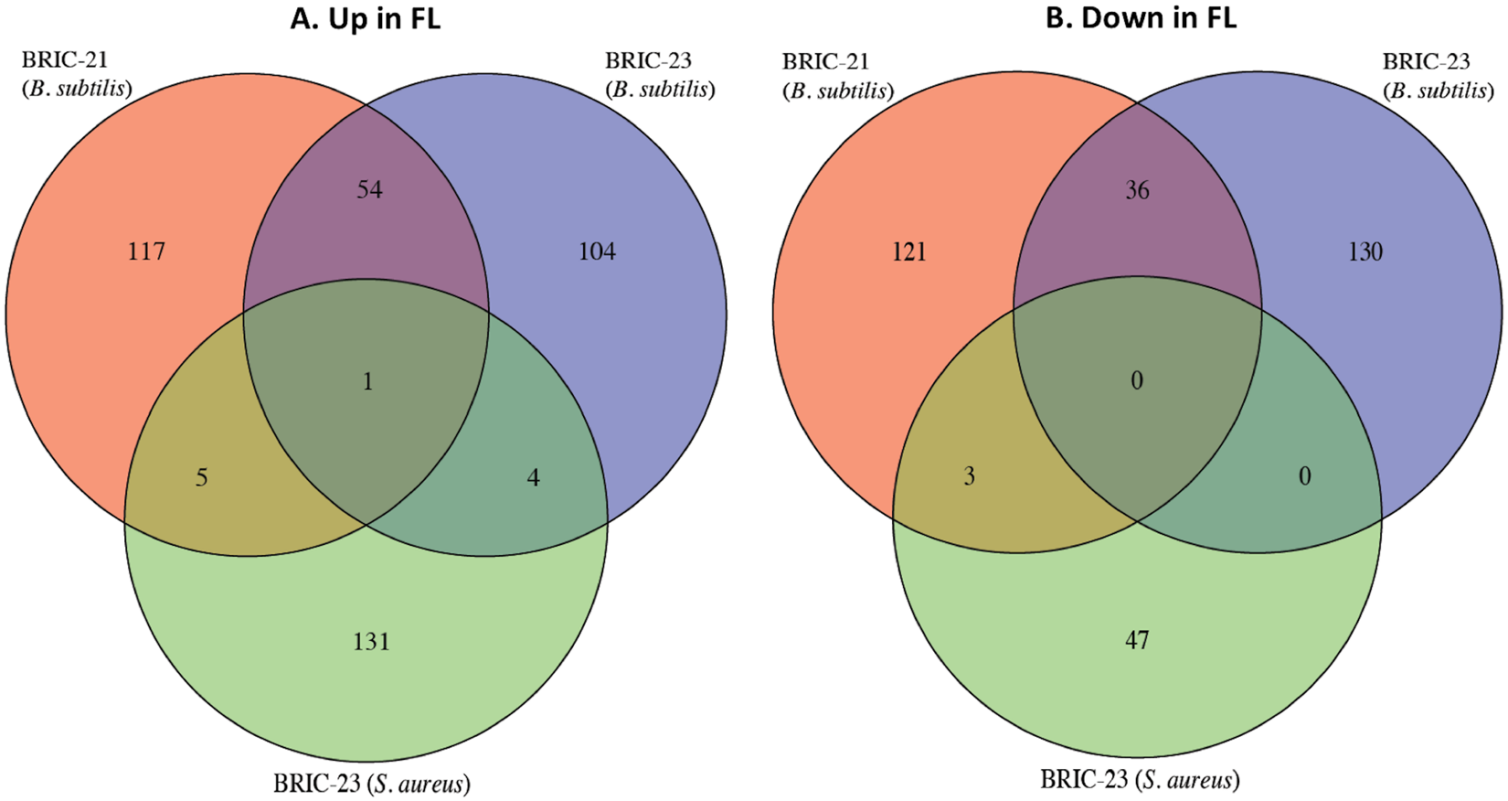
Venn diagrams depicting genes found to be up-regulated (**A**) or down-regulated (**B**) in common among the BRIC-21 and BRIC-23 (both *B. subtilis*) and BRIC-23 (*S. aureus*) spaceflight datasets.

Datasets for Gram-positive species included the Firmicutes *B. subtilis* (BRIC-21 and BRIC-23 missions) and *S. aureus* (BRIC-23 mission) (Table 1). It should be noted here that all three missions were performed using the same hardware (BRIC-PDFU), medium (liquid TSYG), and transcriptome mapping method (RNA-seq), thus minimizing these potential confounding factors^15^. The three raw datasets were run through our bioinformatics pipeline and compared to identify differentially expressed genes in common (Fig. 4). *B. subtilis* samples had the highest number of shared differentially expressed genes between two datasets. We identified 55 up-regulated and 36 down-regulated genes in common, resulting in a 33% concordance between the *B. subtilis* BRIC-21 and BRIC-23 datasets. Detailed analysis of the differentially expressed *B. subtilis* genes in the BRIC-21 and BRIC-23 missions will be described in detail in a separate publication (Morrison *et al*., submitted).

**Figure 5:**
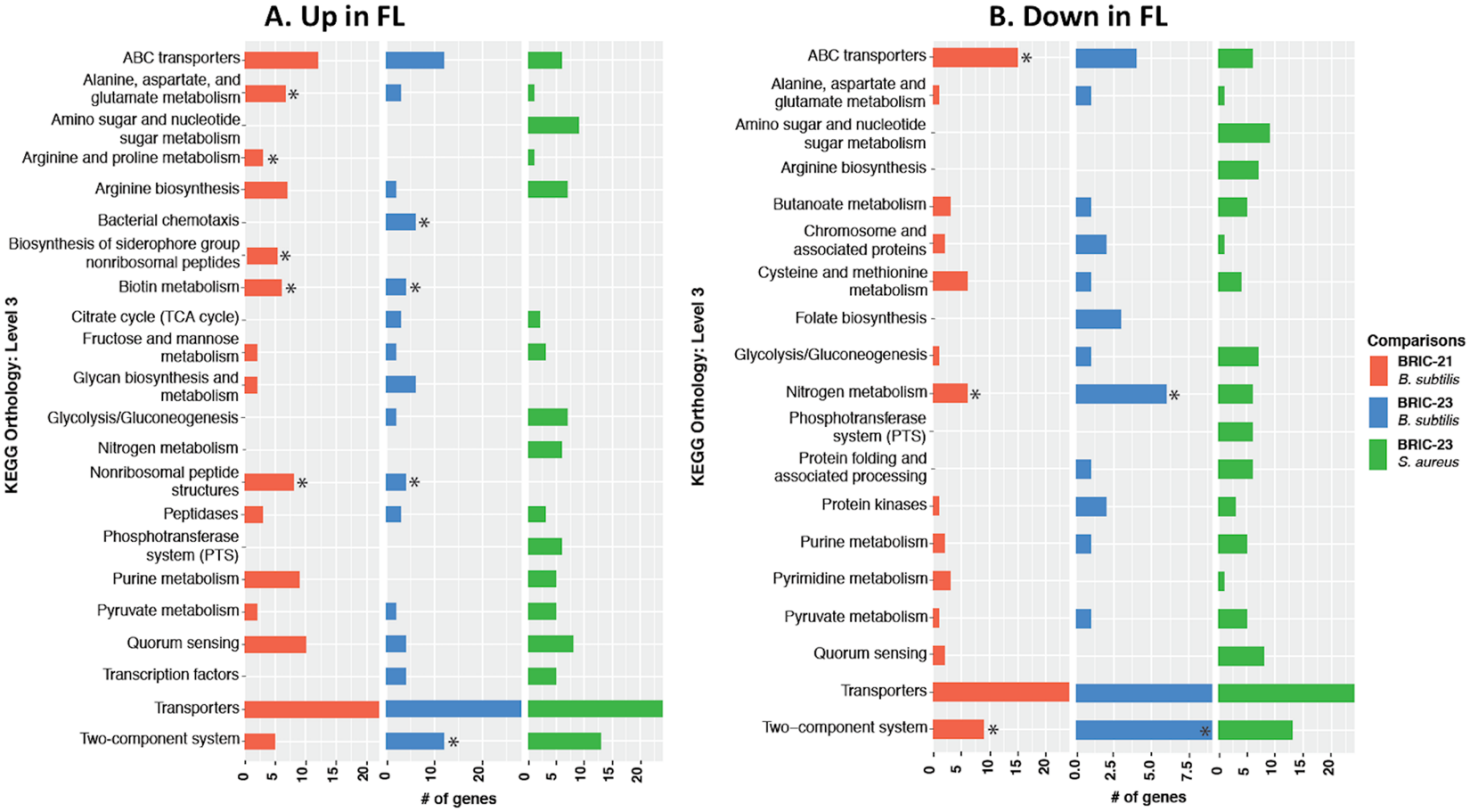
Functional characterization of the differentially expressed genes identified in the datasets for Gram-positive organisms. Depicted are numbers of genes belonging to the indicated biological pathways (KEGG Orthologies) found to up-regulated (**A**) or down-regulated (**B**) in FL samples from the *B. subtilis* BRIC-21 (red bars) and BRIC-23 (blue bars) experiments, and the *S. aureus* BRIC-23 (green bars) experiment. Asterisks denote pathways deemed to be significantly enriched within the up- or down-regulated genes (adjusted *P* < 0.05, Fisher’s exact test).

KEGG Orthology comparisons identified only one gene, KEGG Orthology K00318 annotated as proline dehydrogenase encoded by the *putB* gene^16^, as being significantly up-regulated during spaceflight in all three experiments. In pairwise comparisons, 25 *S. aureus* genes were found to be differentially expressed in at least one of the *B. subtilis* spaceflight experiments (Table 4). Eight genes were significantly down-regulated in both *B. subtilis* datasets, but significantly up-regulated in the *S. aureus* datasets, including nitrate reductase (*narG, narH, narJ*), nitrite reductase (*nasD, nasE*), arginosuccinate synthase (*argG*), lactate dehydrogenase (*ldh*) and lactate permease (*lctP*) (Table 4). Functional enrichment analysis of the Gram-positive datasets found significant enrichment of 3 up-regulated KEGG pathways only in *B. subtilis* BRIC-21 FL samples (the pathways for alanine, aspartate, and glutamate metabolism; arginine and proline metabolism; and siderophore biosynthesis) (Fig. 5A). *B. subtilis* BRIC-23 FL samples were significantly enriched in up-regulated chemotaxis and two-component systems, and the up-regulated KEGG pathways for biotin metabolism and non-ribosomal peptide antibiotic biosynthesis were significantly enriched in both *B. subtilis* BRIC-21 and BRIC-23 experiments (Fig. 5A). No up-regulated pathways were significantly enriched in the *S. aureus* BRIC-23 experiment (Fig. 5A). Regarding down-regulated pathways, functional enrichment analysis identified significant enrichment of ABC transporters in the *B. subtilis* BRIC-21 dataset, and the pathways of nitrogen metabolism and two-component systems in both the *B. subtilis* BRIC-21 and BRIC-23 datasets (Fig. 5B). Again, no down-regulated pathways were significantly enriched in the *S. aureus* BRIC-23 experiment (Fig. 5B).

**Table 4:** KEGG Orthology (KO) numbers identified as differentially expressed in two or more datasets using Gram-positive organisms*.

KO	*B. subtilis*				*S. aureus*		Function
	BRIC-21		BRIC-23		BRIC-23		
	L2FC	P value	L2FC	P value	L2FC	P value	
K00016	**−3.609**	**3.18E-06**	**−2.313**	**1.27E-04**	**1.019**	**2.39E-03**	L-lactate dehydrogenase
K00318	**3.671**	**8.84E-05**	**1.016**	**4.39E-04**	**1.06**	**1.51E-08**	proline dehydrogenase
K00362	**−2.656**	**2.86E-04**	**−2.016**	**3.27E-06**	**2.076**	**4.83E-06**	nitrite reductase (NADH) large subunit
K00363	**−1.661**	**4.51E-04**	**−1.202**	**1.63E-07**	**2.013**	**4.74E-05**	nitrite reductase (NADH) small subunit
K00370	**−3.405**	**7.22E-06**	**−1.951**	**8.86E-04**	**1.537**	**2.42E-05**	nitrate reductase/nitrite oxidoreductase, alpha subunit
K00371	**−3.32**	**1.31E-05**	**−2.081**	**2.74E-04**	**1.284**	**8.85E-04**	nitrate reductase/nitrite oxidoreductase, beta subunit
K00373	**−3.012**	**3.89E-05**	**−2.149**	**1.02E-04**	**1.113**	**3.19E-03**	nitrate reductase molybdenum cofactor assembly chaperone
K00609	**−3.307**	**9.93E-04**	−0.379	0.458	**−1.621**	**9.09E-06**	aspartate carbamoyltransferase catalytic subunit
K00611	**1.914**	**8.87E-05**	0.066	0.585	**3.343**	**2.72E-06**	ornithine carbamoyltransferase
K01465	**−3.142**	**1.62E-03**	−0.174	0.738	**−1.618**	**2.22E-05**	dihydroorotase
K01940	**2.607**	**3.97E-06**	**1.3**	**1.39E-06**	**−1.035**	**1.98E-04**	argininosuccinate synthase
K01955	**3.995**	**5.38E-06**	0.776	4.74E-04	**−1.633**	**4.39E-05**	carbamoyl-phosphate synthase large subunit
K01956	**3.39**	**1.62E-05**	0.174	0.44	**−1.628**	**4.36E-05**	carbamoyl-phosphate synthase small subunit
K02824	**−2.491**	**0.0011**	−0.117	0.698	**−1.444**	**2.83E-05**	uracil permease
K03303	**−3.351**	**5.97E-07**	**−1.775**	**3.73E-04**	**1.656**	**6.87E-06**	lactate permease
K03758	−0.043	0.858	**1.083**	**1.13E-05**	**3.111**	**2.80E-06**	arginine:ornithine antiporter/lysine permease
K05338	0.772	0.373	**1.491**	**3.09E-03**	**1.079**	**0.0197**	holin-like protein
K05339	0.628	0.397	**1.688**	**7.30E-04**	**1.348**	**1.13E-04**	holin-like protein LrgB
K05845	**1.594**	**1.67E-04**	0.318	0.065	**1.181**	**1.58E-06**	osmoprotectant transport system substrate-binding protein
K05846	**1.815**	**2.69E-05**	0.45	0.014	**1.116**	**1.01E-05**	osmoprotectant transport system permease protein
K05847	**1.559**	**7.26E-05**	0.374	0.048	**1.151**	**6.19E-09**	osmoprotectant transport system ATP-binding protein
K05847	**1.801**	**5.40E-06**	0.417	0.022	**1.166**	**8.69E-07**	osmoprotectant transport system ATP-binding protein
K06518	−0.706	0.139	**1.24**	**2.26E-07**	**2.118**	**3.51E-10**	holin-like protein
K16264	1.137	0.062	**3.015**	**4.14E-06**	**1.54**	**6.62E-10**	cobalt-zinc-cadmium efflux system protein
					**1.34**	**2.98E-11**	zinc resistance protein

*Significantly differentially expressed transcripts are denoted in boldface type.

### Gene Set Enrichment Analysis

The analytical method used above (i.e., differential expression analysis of single genes) failed to uncover any commonalities in the response of all bacteria tested to the spaceflight environment. However, because these organisms belong to various taxa which have evolved divergent mechanisms for dealing with stress, it is possible that commonalities in their response to spaceflight might not be revealed by single-gene analyses. Therefore, we turned to Gene Set Enrichment Analysis (GSEA), an analytical method that derives its power by focusing on groups of genes that share common biological functions, chromosomal locations, or regulation^17^.

### GSEA of Gram-negative datasets

**Figure 6:**
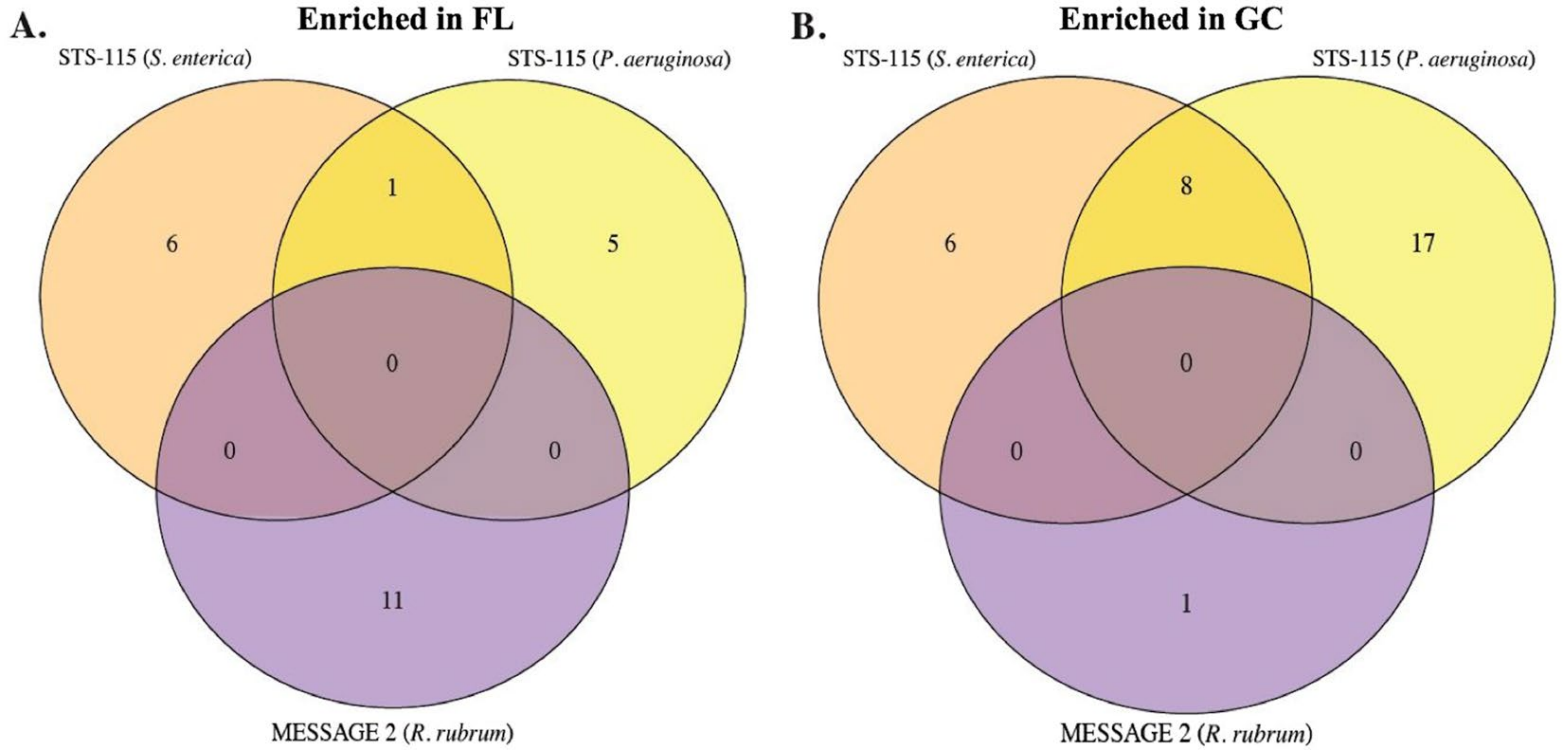
Venn diagrams depicting KEGG gene sets found to be enriched in FL (**A**) or GC (**B**) samples in common among the MESSAGE 2 (*R. rubrum*), STS-115 (*S. enterica*), and STS-115 (*P. aeruginosa*) spaceflight datasets.

GSEA results of the Gram-negative datasets are presented in Table 5 and Fig. 6. Raw microarray fluorescence images were processed and normalized using the same pipeline used for differential expression analysis as described in Methods. Normalized expression value files were converted into the required format for GenePattern and GSEA was performed using the default settings.

**Table 5:** GSEA analysis of spaceflight datasets*.

Dataset	Enriched in FL	Enriched in GC
MESSAGE 2 (*R. rubrum*)	*Ribosome* **Protein export** Oxidative phosphorylation Starch and sucrose metabolism *TCA cycle Glycolysis/Gluconeogenesi*s Carbon metabolism *Butanoate metabolism* *Aminoacyl-tRNA Biosynthesis* Bacterial secretion system Mismatch repair	Nitrotoluene degradation
BASE A (*R. rubrum*)	**Protein export**	none found
STS-115 (*P. aeruginosa*)	*Nitrogen metabolism* Synthesis and degradation of ketone bodies Sulfur metabolism Valine leucine and isoleucine degradation Styrene degradation ***ABC transporters***	***Ribosome*** **Oxidative phosphorylation** **Protein export** **Purine metabolism RNA degradation** Biosynthesis of amino acids *Pyrimidine metabolism* **Carbon metabolism** Pentose phosphate pathway Fatty acid biosynthesis *Homologous recombination* Lysine biosynthesis Lipopolysaccharide biosynthesis **Aminoacyl-tRNA biosynthesis** Methane metabolism Thiamine metabolism Streptomycin biosynthesis Flagellar assembly *Cysteine and methionine metabolism* **TCA cycle** Alanine, aspartate, and glutamate metabolism DNA replication Fatty acid metabolism 2-oxocarboxylic acid metabolism Mismatch repair
STS-115 (*S*. *enterica*)	Pentose and glucuronate interconversions Ascorbate and aldarate metabolism ***ABC transporters*** *Arginine biosynthesis* Benzoate degradation 2-oxocarboxylic acid metabolism *Biosynthesis of siderophore group* *nonribosomal peptides*	***Ribosome*** **Protein export** Bacterial chemotaxis **Oxidative phosphorylation** *Propanoate metabolism* **Carbon metabolism** **Aminoacyl-tRNA biosynthesis** **Purine metabolism** **RNA degradation** Glutathione metabolism *Glycine, serine, and threonine metabolism* **TCA cycle** Bacterial secretion system Pyruvate metabolism
STS-123 (*S. enterica*)	none found	none found
BRIC-21 (*B. subtilis*)	**Biotin metabolism Nonribosomal peptide structures** ***Biosynthesis of siderophore group*** ***nonribosomal peptides*** ***Arginine metabolism*** **Quorum Sensing** Alanine, aspartate, and glutamate metabolism Arginine and proline metabolism ***Aminoacyl-tRNA biosynthesis Ribosome***	**Valine, leucine, and isoleucine biosynthesis** Pantothenate and CoA biosynthesis **Folate biosynthesis** *Glycine, serine, and threonine metabolism* Histidine metabolism *Cysteine and methionine metabolism* C5-branched dibasic acid metabolism
BRIC-23 (*B. subtilis*)	***Ribosome*** ***Aminoacyl-tRNA biosynthesis*** Bacterial chemotaxis ***Biosynthesis of siderophore group*** ***nonribosomal peptides*** **Nonribosomal peptide structures** ***TCA cycle*** *ABC transporters* Flagellar assembly **Biotin Metabolism**	Nitrogen metabolism Peptidoglycan biosynthesis Porphyrin and chlorophyll metabolism **Folate biosynthesis** *Homologous recombination* *Propanoate metabolism* **Valine, leucine, and isoleucine degradation** ***Pyrimidine metabolism***
BRIC-23 (*S. aureus*)	*Nitrogen metabolism* Amino sugar and nucleotide sugar metabolism Riboflavin metabolism **Quorum Sensing** ***TCA cycle*** * Glycolysis/gluconeogenesis* Two-component system ***Arginine biosynthesis*** *Butanoate metabolism* Pentose phosphate pathway Phosphotransferase system Pyruvate metabolism Chloroalkane and Chloroalkene degradation	*Ribosome* Biotin metabolism Phenylalanine, tyrosine, and tryptophan biosynthesis ***Pyrimidine metabolism***

*Shown are Level 3 KEGG Orthology gene sets enriched in each dataset. Gene sets denoted in boldface type were also enriched in datasets from organisms of the same Gram-staining group; gene sets in *italic* type were enriched in datasets from organisms of the opposite Gram-staining group.

*P. aeruginosa*. Roy *et al*.^18^ previously conducted a GSEA on the STS-115 *P. aeruginosa* dataset. Their *P. aeruginosa* GSEA identified 36 enriched KEGG gene sets (8 enriched in FL and 28 enriched in GC), whereas our GSEA identified 31 enriched gene sets (6 enriched in FL and 25 enriched in GC), for a concordance rate of 60% between our results and those of Roy *et al*.^18^.

*S. enterica*. Roy *et al*.^18^ also performed a GSEA on the STS-115 and STS-123 *S. enterica* datasets. For the STS-115 experiment Roy *et al*. reported 19 enriched KEGG gene sets (1 enriched in FL and 18 enriched in GC samples). Our bioinformatic normalization and GSEA identified 21 enriched KEGG gene sets (7 in FL and 14 in GC), and a pair-wise comparison of the results resulted in a 38% concordance between the two GSEA results. GSEA of the STS-123 experiment by Roy *et al*. reported a total of 13 enriched KEGG gene sets (3 enriched in FL and 10 enriched in GC)^18^. However, when the STS-123 data was processed using our pipeline, GSEA found no enriched KEGG gene sets in FL or GC samples (Table 5).

*R. rubrum*. GSEA of the MESSAGE 2 experiment identified 13 enriched KEGG gene sets (12 enriched in FL and 1 enriched in GC) (Table 5 and Fig. 6). GSEA of the BASE A experiments identified only 1 enriched KEGG gene set in the FL samples and no enriched gene sets in the GC samples (Table 5). Pairwise comparisons of the two *R. rubrum* experiments found that the KEGG gene set “protein export” was enriched in both MESSAGE 2 and BASE A experiments (Table 5), suggesting a possible organism-specific response.

Cross-comparison of Gram-negative datasets revealed no gene sets that were enriched in all datasets (Fig. 6). Pairwise comparisons of the datasets found 9 KEGG enriched gene sets (1 in FL and 8 in GC) (Table 5) in the STS-115 *P. aeruginosa* and *S. enterica* experiments (Fig. 6). Pairwise comparisons of the two STS-115 experiments and the two *R. rubrum* experiments did not uncover any common enriched gene sets in FL (Fig. 6A) or GC (Fig. 6B) samples.

### GSEA of Gram-positive datasets

**Figure 7:**
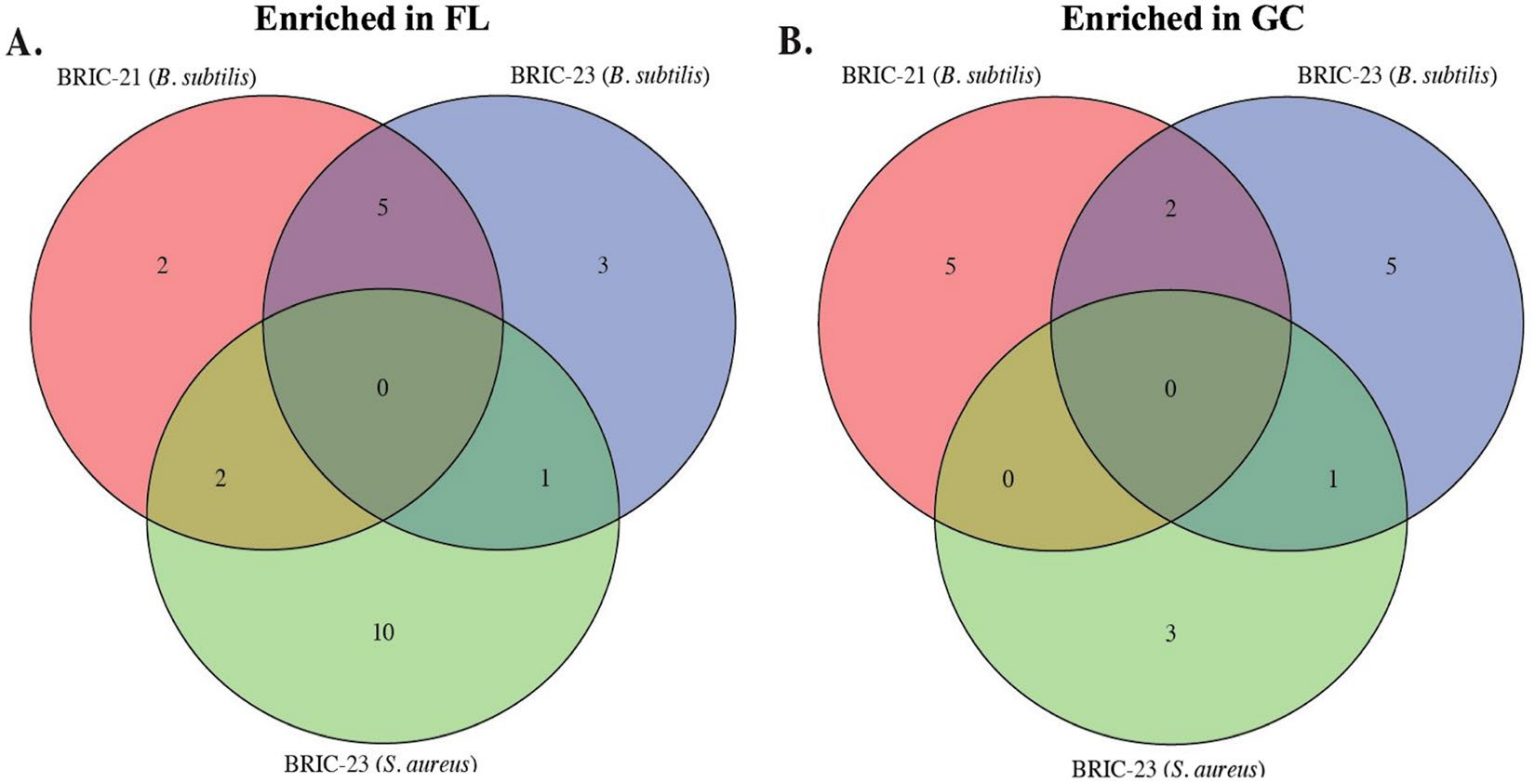
Venn diagrams depicting KEGG gene sets found to be enriched in FL (**A**) or GC (**B**) samples in common among the BRIC-21 and BRIC-23 (both *B. subtilis*) and BRIC-23 (*S. aureus*) spaceflight datasets.

GSEA results of the Gram-positive datasets are presented in Table 5 and Fig. 7. As noted with the Gram-negative datasets, there were no KEGG gene sets that were enriched in all three Gram-positive datasets (Fig. 7). Pair-wise comparisons between the datasets identified a total of 11 shared gene sets (8 enriched in FL and 3 enriched in GC). Seven of these gene sets (5 in FL and 2 in GC) were enriched in both *B. subtilis* datasets resulting in a 27% concordance rate between the two *B. subtilis* experiments (Fig. 7). Two gene sets, “arginine biosynthesis” and “quorum sensing”, were enriched in BRIC-21 *B. subtilis* and BRIC-23 *S. aureus* FL samples (Table 5), and two gene sets, “TCA cycle” in FL and “pyrimidine metabolism” in GC, were enriched in the BRIC-23 *B. subtilis* and *S. aureus* datasets (Table 5).

## Discussion

In order to determine the effect that cultivation in the human spaceflight environment has on global gene expression in bacteria, several transcriptome studies have been performed, and have reported that spaceflight alters the transcript levels of genes involved in primary and secondary metabolism, ribosomal proteins, and virulence factors^8–11,15^. For the most part, these studies were performed independently with little or no direct comparison across different experiments. In this communication we describe the first meta-analysis comparing all publicly available transcriptome profiles from bacteria exposed to the human spaceflight environment. Using a standardized bioinformatics pipeline, the datasets were normalized and analyzed both for differentially expressed single genes and enriched gene sets. Neither single-gene analysis nor GSEA uncovered any genes or gene sets that were significantly differentially expressed across all datasets examined, a finding which does not support the notion of a shared bacterial “spaceflight response” at the level of the transcriptome.

There are several potential reasons why the meta-analysis described here did not uncover a common spaceflight response. First may be due to the disparate collection of bacteria tested, consisting of both Gram-negative and Gram-positive species. It has been well-documented that microbes belonging to widely dispersed taxonomic groups can respond to the same environmental stress using partially overlapping and partially distinct mechanisms^19–21^. A second reason may derive from the diverse experimental setups used; the eight datasets were derived from experiments using 5 different types of growth media (2 semisolid and 3 liquid) and 4 different types of spaceflight hardware (Table 1). Such variation could be visualized by PCA of different datasets utilizing the same organisms (Fig. 1). The highest percentage of explained variance between datasets (PC1) indicated that differences in cultivation conditions (e.g., media, hardware, incubation time) exerted a greater effect on global gene expression than differences in FL vs. GC samples (PC2) (Fig. 1). In most bacterial studies conducted in space, experimenters chose growth conditions and medium according to the historical precedent of the particular bacterium being used. While this is a perfectly valid justification when designing a single experiment, such confounding factors make it difficult to separate spaceflight effects from procedural, hardware, or media-specific effects. Third, the Gram-negative datasets were obtained several years ago using traditional fluorescence microarrays, necessitating conversion of the data into a format compatible with modern RNA-seq technology for statistical comparison. When statistical correction for multiple testing bias was applied, 2 of the 5 Gram-negative datasets (STS-123 *S. enterica* and STS-115 *P. aeruginosa*) demonstrated essentially no statistically significant difference in their transcriptome patterns in FL vs. GC samples. Fourth, the final phenotype that a bacterium would exhibit is the product of its physiological response to the spaceflight environment. Measuring the transcriptome captures only one aspect of physiology; it does not take into account the numerous post-transcriptional processes (translation, protein processing and modification, metabolic regulation of enzyme activity, assembly of supramacromolecular structures, etc.) which must take place in order for a microbe to manifest its final phenotype.

A slightly more consistent situation was encountered in the Gram-positive datasets used in this study, as these 3 experiments were designed and executed using the same liquid medium (TSYG) and hardware (BRIC-PDFU) (Table 1). However, a notable difference in the 3 experiments were the times of incubation, hence growth phase at which cells were frozen for subsequent RNA extraction. According to pre-flight ground validation experiments^15^, BRIC-21 *B. subtilis* samples were grown to late exponential phase, whereas BRIC-23 *B. subtilis* and *S. aureus* samples were more likely experiencing the transition from exponential to stationary phase^15^. By PCA, this difference was revealed in PC1 between the BRIC-21 and BRIC-23 *B. subtilis* samples (Fig. 1A). Cross-comparison of the *B. subtilis* and *S. aureus* datasets found only one gene, proline dehydrogenase, up-regulated in all three experiments, and there were no significantly enriched gene sets common among all 3 datasets (Table 5, Fig. 7). In fact, we noticed that several genes associated with growth under oxygen limitation (encoding nitrate/nitrite reductases and lactate permease) were down-regulated in *B. subtilis* FL samples but up-regulated in *S. aureus* FL samples (Table 4). Oxygen availability should have been consistent across all samples, due to their being cultivated in the same medium (TSYG) and hardware (BRIC-PDFUs); thus it is possible that these differences may be pointing towards organism-specific responses to the spaceflight environment.

Interestingly, we noted that the *B. subtilis* BRIC-21 and BRIC-23 datasets exhibited the highest concordance (~33%) in shared differentially expressed transcripts of any of the datasets examined (Fig. 4). Of all the datasets examined, these two were generated under the closest approximation of identical conditions. This observation reinforces the notion that a necessary first step towards distinguishing true spaceflight effects from experimental noise is the strict control and replication of experimental conditions. In order to develop an accurate understanding of how bacteria respond and adapt to the human spaceflight environment, we suggest that future spaceflight experiments should attempt to utilize standardized experimental conditions, and to perform flight experiments on at least two independent missions^15^. This would greatly reduce experimental noise and, in the long run, more rigorously address the question of whether microorganisms mount common and consistent responses to spaceflight.

## Methods

### Dataset selection

For this study, we investigated transcriptomes from bacterial cultures grown in the human spaceflight environment and their corresponding ground control cultures. Spaceflight experiments and organisms included: MESSAGE 2 and BASE A (*Rhodospirillum rubrum* strain S1H)^11^; STS-115 (*Salmonella enterica* serovar Typhimurium strain χ3339 and *Pseudomonas aeruginosa* strain PAO1)^8,9^; STS-123 (*S. enterica* serovar Typhimurium strain χ3339)^10^; BRIC-21 (*Bacillus subtilis* strain 168)^22,23^; and BRIC-23 (*B. subtilis* strain 168 and *Staphylococcus aureus* strain UAMS-1)^15^. All of the datasets used are openly available through NASA’s GeneLab Data System (GLDS) repository (genelab.nasa.gov) and are listed in Table 1. It should be noted that some of the GLDS datasets also contained transcriptome profiles obtained from simulated microgravity (clinostat) experiments, but only samples exposed to actual spaceflight and their corresponding ground control samples were analyzed in this study. One dataset, GLDS-95^24^ (*Escherichia coli* strain ATCC 4157), was excluded from this study because transcriptome effects caused by antibiotic stress could not be distinguished from possible effects of spaceflight.

### Microarray processing and normalization

All datasets were preprocessed and normalized individually. Non-Affymetrix microarray files were imported and normalized using the R package *limma*^25^. The *normexp* method^26^ with an offset of 50 was used for background correction, and expression values were normalized within and between arrays using the loess and quantile^27^ methods, respectively. Normalization of Affymetrix files was done using a second R package *affy*^28^. Background correction of Affymetrix files was accomplished using the Robust Multichip Average (RMA) algorithm^29^ and normalization of Affymetrix arrays was accomplished using the same quantile method used in *limma*^25^.

### RNA-seq read alignment, quantification, and normalization

Raw Illumina RNA-seq FASTQ files were transferred directly without preprocessing from the GLDS into the University of Florida’s High-Performance Research Computing system HiPerGator v. 2.0 (https://www.rc.ufl.edu/services/hipergator/). Samples were trimmed using FASTQ Trimmer^30^ and the read quality for each file was determined by FASTQC^31^. Reads were aligned to their appropriate genomes using Bowtie2^32^ and read alignment quality was checked using the program SAMstat^33^. Finally, gene count quantification was performed using HTSeq^34^. The trimmed mean of M-values (TMM) normalization of gene counts was performed in *limma*^25,35^, and the normalized counts were transformed using the built-in ‘voom’^36^ conversion. Reference genomes and annotation files used throughout the RNA-seq analysis were acquired from the National Center for Biotechnology Information Genome page (https://www.ncbi.nlm.nih.gov/genome/).

### Principal Component Analysis

Normalized microarray expression values and RNA-seq gene counts derived from the same organism were combined for principal component analysis (PCA). PCA was performed on each organism individually using the built-in *stat* package in R^37^. The calculated loading scores and explained variance for the first two principal components were plotted in R to visualize sample clustering.

### Differential Expression Analysis

Normalized microarray expression values and RNA-seq gene counts were run through *limma* to identify differentially expressed genes. For a gene to be considered differentially expressed, it had to exhibit at least a 2-fold change with a *P* value < 0.05 between flight (FL) and ground (GC) samples. To reduce false positive results, *P* values were adjusted in *limma* using the Benjamini-Hochberg method^12^. Differentially expressed genes were annotated using the NCBI database. Functional enrichment analysis of differentially expressed genes was carried out using the STRING database^38^.

### Comparison of Differentially Expressed Genes

To compare the differentially expressed genes found in each dataset we used a method based on functional similarity. Kyoto Encyclopedia of Genes and Genomes (KEGG) Orthology (KO) identifiers are genome annotations within the KEGG database^39,40^ which are assigned according to the molecular function of a gene. KO information for all differentially expressed genes was acquired from the Universal Protein Resource (UniProt)^41^. KO numbers shared between multiple datasets were collected for manual inspection. Common KO numbers with opposing magnitude directions (i.e. up in FL for dataset A and down in FL for dataset B) were not considered similar responses to spaceflight and were left out when discussing common responses between differing datasets.

### Gene Set Enrichment Analysis

Gene set enrichment analysis (GSEA) was conducted using the multitool platform GenePattern^42^ following a previously published protocol comparing datasets GLDS-11 and GLDS-15^18^. The gene sets for all organisms used in this study were obtained from the KEGG database. Normalized microarray expression values and RNA-seq gene counts were converted into the format recommended by GenePattern, and GSEA was performed using the default settings. Gene sets with a *P* value < 0.05 and *q*-value < 0.25 were considered to be enriched. Enriched gene sets among FL and GC samples were compared across datasets to identify any similarities.
